# The genomic architecture and association genetics of adaptive characters using a candidate SNP approach in boreal black spruce

**DOI:** 10.1186/1471-2164-14-368

**Published:** 2013-06-01

**Authors:** Julien Prunier, Betty Pelgas, France Gagnon, Mireille Desponts, Nathalie Isabel, Jean Beaulieu, Jean Bousquet

**Affiliations:** 1 Canada Research Chair in Forest and Environmental Genomics, Centre for Forest Research, and Institute for Systems and Integrative Biology, Université Laval, Québec, Québec G1V 0A6, Canada; 2 Natural Resources Canada, Canadian Forest Service, Laurentian Forestry Centre, 1055 rue du PEPS CP 10380 Succ Sainte-Foy, Québec, Québec, G1V 4C7, Canada; 3 Ministère des Ressources Naturelles du Québec, 880 Chemin Sainte-Foy, Québec, Québec, G1S 4X4, Canada; 4 Natural Ressources Canada, Canadian Forest Service, Canadian Wood Fibre Centre, 1055 rue du PEPS CP 10380 Succ Sainte-Foy, Québec, Québec, G1V 4C7, Canada

## Abstract

**Background:**

The genomic architecture of adaptive traits remains poorly understood in non-model plants. Various approaches can be used to bridge this gap, including the mapping of quantitative trait loci (QTL) in pedigrees, and genetic association studies in non-structured populations. Here we present results on the genomic architecture of adaptive traits in black spruce, which is a widely distributed conifer of the North American boreal forest. As an alternative to the usual candidate gene approach, a candidate SNP approach was developed for association testing.

**Results:**

A genetic map containing 231 gene loci was used to identify QTL that were related to budset timing and to tree height assessed over multiple years and sites. Twenty-two unique genomic regions were identified, including 20 that were related to budset timing and 6 that were related to tree height. From results of outlier detection and bulk segregant analysis for adaptive traits using DNA pool sequencing of 434 genes, 52 candidate SNPs were identified and subsequently tested in genetic association studies for budset timing and tree height assessed over multiple years and sites. A total of 34 (65%) SNPs were significantly associated with budset timing, or tree height, or both. Although the percentages of explained variance (PVE) by individual SNPs were small, several significant SNPs were shared between sites and among years.

**Conclusions:**

The sharing of genomic regions and significant SNPs between budset timing and tree height indicates pleiotropic effects. Significant QTLs and SNPs differed quite greatly among years, suggesting that different sets of genes for the same characters are involved at different stages in the tree’s life history. The functional diversity of genes carrying significant SNPs and low observed PVE further indicated that a large number of polymorphisms are involved in adaptive genetic variation. Accordingly, for undomesticated species such as black spruce with natural populations of large effective size and low linkage disequilibrium, efficient marker systems that are predictive of adaptation should require the survey of large numbers of SNPs. Candidate SNP approaches like the one developed in the present study could contribute to reducing these numbers.

## Background

Adaptation to temperature is a major ecological concern in the context of current and forthcoming global warming. Extant species have survived past climate changes by migration or adaptation [1,2]. However, questions have been raised regarding the ability of tree species to cope with predicted rapid climate changes, given their long generation times. In some cases, wildlife managers may have to assist migration by using pre-adapted seedlings if they wish to avoid some species extirpation [3,4]. A better knowledge of the genomic architecture and the identification of genetic polymorphisms that are involved in trait variations related to climate adaptation could help monitor changes in present gene pools while climate change is taking place, and assist tree breeders and forest managers in identifying seedlings that are best adapted to future climatic conditions. As trees play important ecological roles as foundation species [5], their local maintenance or assisted migration might become one of the most important aspects of forest management during the next century.

Methods that are available for identifying polymorphisms related to adaptation fall under two general classes, which are known as the bottom-up and top-down approaches [6]. The first approach identifies putative adaptive markers and genes by searching for deviations from neutral expectation, which would represent signatures of selection. Even though these methods have several advantageous features such as not requiring the identification of the adaptive syndrome [7], they often have been criticised for their difficulty in building realistic demographic models to test, in avoiding single-locus false positives or in estimating their relevance to fitness without phenotypic information. In addition, the possibility of anonymous loci in linkage disequilibrium (LD) with the true selected sites could not be ruled out using such methods [6,8]. Nevertheless, this approach has been successfully applied to a number of tree species for instance, including white spruce, *Picea glauca* (Moench) Voss [9,10], and black spruce, *Picea mariana* (Mill.) BSP [11,12], typically canopy dominant trees in the North American boreal forest.

In contrast, top-down approaches are based on the analysis of the co-segregation of genotype and phenotype. Hence, these approaches appear more convincing than bottom-up methods, since the former associate polymorphisms with adaptive trait variation in individuals that are typically raised in a common garden setting. In its simplest form, top-down methods can be applied to controlled crosses between parents showing variation in adaptive traits, thereby allowing the identification of genomic regions that are involved in adaptive trait variation, i.e., quantitative trait loci (QTL) mapping. This approach has already been successfully used to identify regions involved in adaptive trait variation in white spruce [13], a congeneric species that is largely sympatric with black spruce. Notably, the aforementioned study with white spruce has shown that QTLs for adaptive traits are numerous, with each controlling a small portion of phenotypic variation. In addition, the top-down approach can be applied to individuals that originate from natural populations in order to identify polymorphisms involved in adaptive trait variation in a context where linkage disequilibrium is much lower, as is the case with conifers [10]. Known as genetic association testing, this approach implies that a large set of individuals can be assessed to conduct such identifications [14,15]. Despite their high cost, these tests remain feasible when using a limited list of candidate genes likely involved in genetic adaptation, in order to reduce the number of polymorphisms to be tested. By focusing on gene SNPs, such analyses could result in greater rewards than would randomly located anonymous markers, given their potential direct effects on transcript levels or on amino acid sequences and protein conformations, potentially in relation to variation in adaptive traits [9,16]. Association studies have been successfully used on tree species [15,17-19], but those that are based on gene SNPs have been scarcely performed with individuals well beyond the seedling stage. However, Beaulieu and colleagues [20] identified gene SNPs in relation to mature growth and wood traits in 30-year-old *P. glauca* trees. In doing so, they demonstrated that several hundred candidate genes would need to be tested. Without the means of selecting SNPs that are potentially involved in trait variation, genome scans using SNPs would involve the screening of many more SNPs than there are candidate genes, a condition which is rarely attained for non-model species when large numbers of candidate genes are involved. In addition, implicating all known SNPs tends to increase the rate of false positives while involving SNPs that are potentially in linkage disequilibrium. Therefore, *a priori* filtering of SNPs that is based on criteria, such as their possible involvement in the trait variation being investigated, represents promising candidate SNP approaches that need to be developed, especially for non-model organisms where genotyping costs may represent a substantial consideration.

*Picea mariana* is a widely distributed conifer of the North American boreal forest [21], with its range currently extending from the Atlantic to the Pacific coasts, mostly in Canada and Alaska. Given ongoing trends in climate change, this species is expected to be constrained to a much narrower area in the future, and see its natural range substantially shifting northward [22]. In boreal conifers, earlier budset timing is generally related to better cold resistance [23]. In natural populations of *P. mariana*, provenances from colder regions are characterised by earlier budset timing, as indicated from common garden studies, highlighting the genetic component of variation in this quantitative trait [24,25]. Significant population differentiation for this trait, together with that of tree height, has already been reported [25,26]. Other common garden studies in the largely sympatric *P. glauca* have also shown that budset timing and growth characters are moderately to highly differentiated among populations with respect to variation in geoclimatic factors [27-29]. Therefore, budset timing and height growth are two desirable traits that should be considered in QTL mapping and genetic association tests to identify genetic polymorphisms that are potentially involved in adaptation in boreal spruces.

The first objective of the study was to investigate the genomic architecture of adaptive traits in *P. mariana* by conducting QTL mapping for budset timing and height growth. Mapping allowed us to identify 22 unique regions that were distributed along the genome and explaining, on average, 8.3% of trait variance. After defining a limited list of candidate gene SNPs that are likely involved in adaptive variation, genetic association tests conducted for budset timing and tree height at various ages revealed that 65% of the tested polymorphisms were involved in trait variation. Each significantly associated polymorphism explained a low proportion of trait variance, even though 50% exhibited significant correlations between allele frequency variation and climatic factors of population origins.

## Results

### QTL mapping analyses

**Figure 1:**
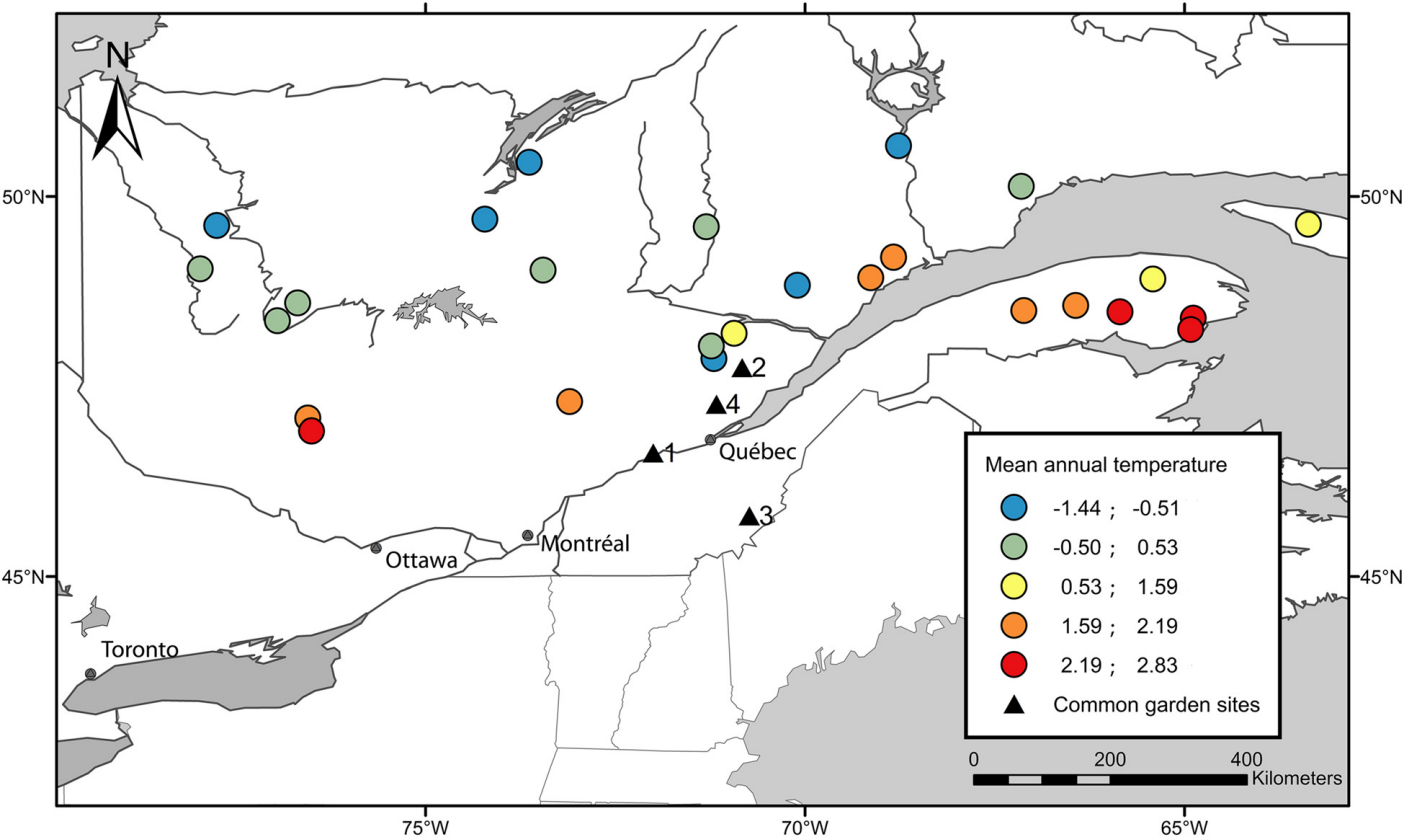
**Locations and mean annual temperatures of geographical origins of *P. mariana *populations that were used in the genetic association study, and locations of sites for the QTL study (common garden sites #1 and #2) and for the provenance/progeny test used in the genetic association study (common garden sites #3 and #4).**

**Figure 2:**
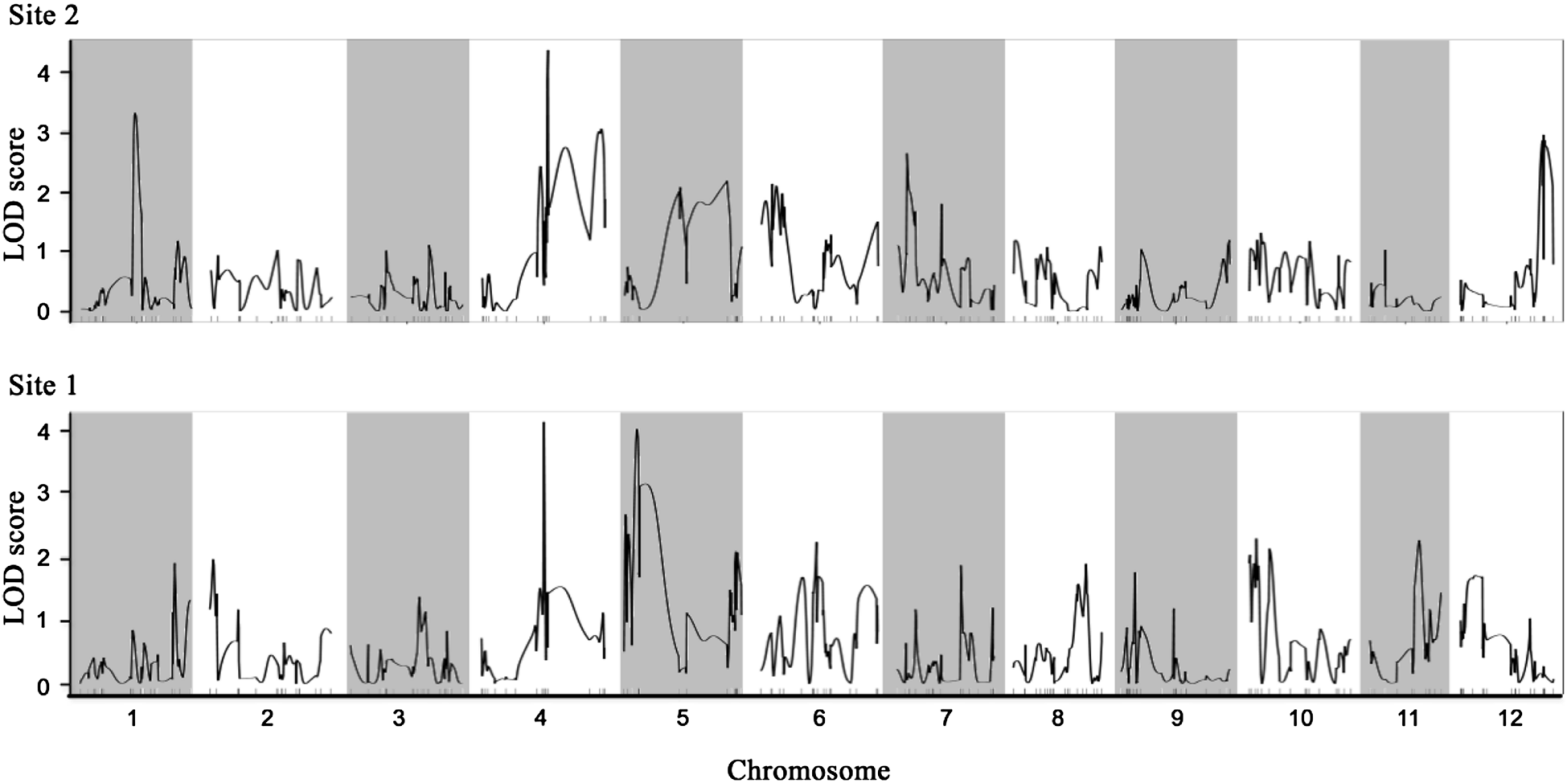
**Example of QTL mapping results for budset timing using non-parametric interval mapping at year 7 for both study sites (#1 and #2). **The complete QTL mapping results are tabulated in Table 1.

Over two years and two climatically different sites (sites #1 and #2, Figure 1), budset timing and height growth were measured in a backcross family that had been previously used to map the genome of black spruce [30,31]. Given that several adaptive traits were not normally distributed, both parametric and non-parametric QTL mapping approaches were used to delineate genomic regions involved in adaptive trait variation. Over all methods and traits, 35 QTLs that were related to growth and budset timing were detected (QTLs results for each trait are tabulated in Table 1). Among these QTLs, 29 were related to budset variation and 8 were related to growth variation (see Figure 2 as an example of R/qtl results for non-parametric interval mapping). Using the non-parametric interval mapping method (nIM), only one QTL was significant at the 5% genome-wide scale for budset variation. In addition, 4 and 2 QTLs were significant at the 5% chromosome-wide scale for budset and growth variation, respectively. Using interval mapping (IM) with transformed data to fit a normal distribution, 3 and 2 additional QTLs were disclosed as significantly involved in budset and growth variation, respectively, at the 5% chromosome-wide scale. The proportion of variance that was explained (PVE) by a single QTL was low, averaging 8.3% and never exceeding 12%. Assuming an additive model among QTL effects for each trait, the total PVE for one trait varied from 5.2 to 47.9%. However, given the potential complexity of QTL interactions, these values might represent overestimates of the QTL combined effects. Among QTLs, 27 were repeatedly involved in quantitative trait variation over sites, years, or methods (Table 1). Though not implicating a direct role in QTL control, 39 mapped genes were located in QTLs that were found to be significantly involved in quantitative trait variation among the progeny (24 mapped genes located in “budset” QTLs, 5 located in “height” QTLs, and 10 involved in both traits).

**Table 1:** **List of the location and characteristics of QTLs found using the three methods in *P. mariana***

**Linkage group **^**a**^	**Trait**	**Year**	**Method**^**b**^	**Site **^**c**^	**QTL**			**LOD threshold**	
					**Position**^**d**^	**LOD score or *P*-value**^**e**^	**PVE **^**f**^	**At linkage group scale**	**At genome-wide scale**
1	growth	2007	IM	site#1	60-73	3.25	6.5	3.4 (2.9)	4.3 (3.8)
1	growth	2006	nIM	site#1	60-78	3.7	-	3.2 (2.8)	4.4 (3.9)
1	budset	2006	IM	site#1	68-73	2.87	7.9	3.3 (2.8)	4.4 (3.7)
1	budset	2007	IM	site#1	81-90	3.25	6.8	3.2 (2.6)	5.1 (3.9)
1	budset	2006	KW	site#2	90.00	****	-	-	-
1	budset	2007	IM	site#1	58-114	3.09	6.5	3.2 (2.8)	4.8 (3.5)
1	budset	2007	nIM	site#1	108-108	3.1	-	3.2 (2.8)	4.4 (3.9)
1	budset	2007	K-W	site#1	149.00	******	-	-	-
3	budset	2007	K-W	site#2	85.00	***	-	-	-
3	growth	2007	K-W	site#2	85.00	***	-	-	-
4	budset	2007	K-W	site#1	76.00	****	-	-	-
4	budset	2007	K-W	site#1	87.00	***	-	-	-
4	budset	2007	nIM	site#2	89-89	4.57	-	3.2 (2.8)	4.5 (4.2)
4	budset	2007	nIM	site#1	75-118	4.1	-	3.6 (2.8)	4.4 (3.9)
4	budset	2006	nIM	site#2	88-119	3.42	-	3.3 (2.7)	4.7 (4.1)
4	budset	2006	K-W	site#2	156.00	****	-	-	-
4	budset	2006	K-W	site#2	160.00	****	-	-	-
4	budset	2006	K-W	site#2	162.00	****	-	-	-
5	budset	2006	K-W	site#1	18.00	****	-	-	-
5	budset	2006	K-W	site#1	19.00	****	-	-	-
5	budset	2006	K-W	site#1	20.00	***	-	-	-
5	budset	2006	nIM	site#1	17-22	4.2	-	3.1 (2.8)	4.5 (4.1)
5	budset	2006	nIM	site #1	36-42	3.2	-	3.1 (2.8)	4.5 (4.1)
5	budset	2006	IM	site #1	34-68	4.47	5.3	4.3 (4.0)	4.5 (3.4)
5	budset	2006	IM	site #1	59-65	3.17	9.7	3.4 (2.9)	4.4 (3.7)
5	growth	2006	IM	site #1	111-129	3.55	7.8	3.4 (2.9)	5.1 (4.0)
5	budset	2006	K-W	site #2	131.00	***	-	-	-
5	budset	2006	K-W	site #1	131.00	***	-	-	-
5	budset	2007	IM	site #1	127-134	3.43	6.1	3.6 (3.0)	4.8 (3.5)
5	growth	2006	IM	site #1	131-142	3.2	7.6	3.5 (3.0)	4.5 (3.8)
5	budset	2006	nIM	site #2	142-142	2.7	-	3.0 (2.7)	4.7 (4.1)
5	growth	2007	IM	site #1	140-147	3.43	6.6	3.3 (2.9)	4.3 (3.8)
6	budset	2006	IM	site #2	0-15	2.99	11.3	3.0 (2.5)	4.2 (3.7)
6	budset	2006	IM	site #2	5-30	2.99	11.3	3.2 (2.7)	4.3 (3.9)
6	budset	2006	nIM	site #1	82-86	2.7	-	3.0 (2.5)	4.5 (4.1)
6	budset	2007	nIM	site #2	137-147	3.7	-	2.9 (2.4)	4.5 (4.2)
6	budset	2007	K-W	site #2	169.00	*****	-	-	-
6	budset	2007	K-W	site #2	170.00	****	-	-	-
7	budset	2006	nIM	site #2	11-16	2.8	-	3.0 (2.7)	4.7 (4.1)
7	growth	2006	IM	site #1	82-95	2.89	7.5	3.2 (2.7)	5.1 (4.0)
7	growth	2006	nIM	site #1	99-99	2.6	-	3.1 (2.6)	4.4 (3.9)
8	budset	2007	IM	site #2	9-15	2.99	10.8	3.1 (2.6)	4.2 (3.8)
8	budset	2007	IM	site #2	18-29	3.14	11.7	3.1 (2.8)	4.8 (4.1)
9	growth	2007	IM	site #2	62-70	2.95	12.3	3.0 (2.7)	5.3 (4.0)
10	budset	2006	K-W	site #1	9.00	***	-	-	-
10	budset	2006	K-W	site #1	20.00	***	-	-	-
10	budset	2007	nIM	site #1	99-104	3	-	3.3 (2.9)	4.4 (3.9)
11	budset	2007	IM	site #2	0-5	2.94	9.9	2.0 (1.6)	4.2 (3.8)
11	budset	2007	nIM	site #2	7-14	2.3	-	2.6 (2.1)	4.5 (4.2)
12	budset	2006	nIM	site #1	11-11	2.7	-	3.1 (2.7)	4.5 (4.1)
12	growth	2007	nIM	site #1	31-33	3.8	-	3.1 (2.8)	4.6 (3.9)
12	growth	2007	IM	site #1	37-48	3.22	8.2	3.1 (2.7)	4.3 (4.0)
12	budset	2006	IM	site #2	95-106	2.98	4	2.9 (2.5)	4.3 (3.9)
12	budset	2006	nIM	site #2	98-100	2.9	-	3.2 (2.8)	4.7 (4.1)
12	budset	2006	K-W	site #2	107.00	***	-	-	-
12	budset	2006	K-W	site #2	109.00	****	-	-	-

^a ^Linkage group according to Pavy *et al.* 2008.

^b ^Methods of QTL detection: nIM, non-parametric interval mapping; IM, interval mapping; K-W, Kruskal-Wallis test (see material and methods).

^c ^According to Figure 1 and Additional file 1: Table S1.

^d ^Position in cM according to Kosambi function.

^e ^p-value for the Kruskal-Wallis tests: *** > 0.995; **** > 0.999; ***** > 0.9995; ****** > 0.9999.

^f^ PVE: proportion of variance explained.

### Genetic association tests

#### Candidate gene polymorphisms and genotyping success

Candidate gene SNPs were identified from results that had been obtained in a previous outlier study related to climate variation [26] and from comparisons among gene sequences of individuals exhibiting extreme adaptive phenotypes (see methods). Out of 583 SNPs that were tested (from 313 genes), the outlier detection study [26] identified 26 SNPs (from 25 genes) under putative divergent selection. These SNPs harboured differentiation indices (*F*_ST_) among climatic population partitions significantly higher than that expected under a neutral model of evolution. Consequently, they were retained as candidate SNPs for the present genetic association tests. Additionally, DNA sequence comparisons for 434 genes among pools of individuals with extreme phenotypic values for budset timing or tree height revealed a total of 41 SNPs segregating between opposite pools (11 segregating between pools for “budset timing,” 18 for “tree height,” and 12 for both characters), representing as many distinct genes. Because of some overlap, these two methods yielded a total of 52 distinct candidate SNPs that were representative of 51 genes for use in genetic association testing (see below).

A total of 1355 individuals from the two study sites were successfully genotyped for 78 SNPs using Sequenom iPLEX Gold Genotyping technology, including 31 control SNPs and 47 candidate SNPs. These individuals were also genotyped for the 5 remaining candidate SNPs using Taqman SNP Genotyping technology. Of the 1355 successfully genotyped individuals, 917 were from site #3 and 437 from site #4 (Figure 1).

#### Relative kinship and association results

Using the genotypes that were obtained for control SNPs, pairwise relative kinship coefficients (*K*) among individuals were estimated using the method of Loiselle *et al.*[32]. Average kinship among all individuals was *K* =0.0004, indicating a very low degree of relatedness in the sampled populations. In addition, possible hierarchical population structure was tested using the software STRUCTURE [33]. The analysis revealed no population genetic structure in the sampled area, since the highest probability that was obtained was for *k* = 1 group of populations. Such low average kinship (among individuals) and the absence of population structure at the regional scale were not surprising, given that all sampled populations were part of the same glacial lineage with respect to nuclear DNA [12] and cpDNA [34]; also, black spruce is characterised by wind pollination, an outcrossing mating system, and extensive gene flow [35,36].

For the genetic association tests, we applied the method developed by Yu *et al.*[37] that is based on a linear mixed-model and takes into account the degree of genetic relatedness among individuals. To correct for multiple testing, a *q*-value threshold of 10% (FDR [38]) was used to declare significant associations. Using this threshold, none of the control SNPs were significantly associated with trait variations (results not shown). Among the set of candidate SNPs, a total of 34 gene SNPs (representing as many distinct genes) were significantly associated with variation in adaptive traits in black spruce natural populations (Table 2). Twenty-two SNPs were significantly associated with budset variation, while 20 SNPs were significantly associated with height variation. Eight SNPs were involved with both traits, although rarely in the same year, and usually in the following growing seasons (Table 2). The total of 34 significant gene SNPs included 20 and 22 SNPs that were involved in trait variation for site #3 and site #4, respectively, and which were used for association genetic testing; 8 SNPs were detected for the same trait in both sites. In further comparisons of the two sites, no difference could be discerned regarding the average *P*-values or *q*-values of significant SNPs between sites (*t*-test, *P* = 0.45), but sites differed significantly regarding the average estimated values of PVE (proportion of explained variance) (*t*-test, *P* < 0.0001). Indeed, significant SNPs that were detected for site #3, which was situated in milder climatic conditions (Additional file 1: Table S1), rarely explained more than 1% of trait variation, while PVE values for significant SNPs that were detected for site #4, with harsher climatic conditions, frequently exceeded 1%, with several values above 2% (Table 2). When comparing SNPs among years, 11 significant SNPs were corroborated over several years, but fewer SNPs were significantly associated with tree height in the last year of measurement (age 12). Interestingly, data from the final measurement year were not used to conduct bulk segregant analysis that lead to the identification of candidate gene SNPs to be tested (see Methods), suggesting that partially different gene sets are acting at different ages for the same adaptive trait.

**Table 2:** **List and description of gene SNPs significantly associated with adaptive trait variation in *P. mariana***

**SNP**	**Gene ID**^**a**^	**Gene family**	**LG**^**b**^	**Site **^**c**^	**Trait**	**Age**	**p-value**	**PVE**^**d**^	**q-value**	**Correlated climatic factors**^**e**^
*c06511e*	*GQ02808_G21*	AUX/IAA	1	4	budset	7	0.01	1.40	0.06	TWM
*c11176n*	*GQ03816_N20*	Unknown	1	3	budset	7	0.08	0.33	0.08	-
*c16364e*	*GQ04002_G01*	C2H2 zinc finger	1	3	height	12	0.02	2.23	0.10	PWM*
*c02239c*	*GQ03118_J01*	BAM1	3	3	budset	7	0.04	0.11	0.08	PWM***, AAP*
*c03870a*	*GQ0168_K17*	C3HC4 Ring finger	3	4	budset	8	0.02	1.95	0.08	MAT*, TWM***
*c09889a*	*GQ02801_L07*	NAC	3	4	height	7	0.07	0.74	0.10	MAT*, TWM*, TCM*
*c04073m*	*GQ03313_I07*	Zinc ion binding	4	4	budset	7	0.00	2.26	0.03	-
*c05720c*	*GQ03009_F08*	C3HC4 Ring finger	4	4	height	6	0.06	1.29	0.06	-
				3	budset	7	0.06	0.41	0.10	
				3	height	12	0.01	2.36	0.1	
*c10254m*	*GQ0197_M23*	Beta-glucosidase	4	3	budset	7	0.01	0.48	0.05	-
*c02343e*	*GQ03102_D15*	AP2	5	3	budset	7	0.06	0.33	0.08	TWM*, DGD*
*c04662f*	*GQ0011_L04*	AP2	5	4	height	7	0.08	0.73	0.10	-
*c09406f*	*GQ03511_H09*	LEA	5	3	budset	7	0.06	0.41	0.10	TCM*, TWM**
*c09898f*	*GQ02813_D01*	RAP2	5	4	height	6	0.01	2.01	0.03	MAT***, TWM***, TCM*
				4	height	7	0.00	2.53	0.03	
*c10140e*	*GQ04111_I24*	AP2	5	4	budset	7	0.00	2.85	0.01	-
				4	budset	8	0.03	1.70	0.08	
				3	budset	7	0.02	0.24	0.05	
*c12722e*	*GQ03309_B18*	U-box superfamily	5	3	budset	7	0.07	0.11	0.08	-
				3	height	12	0.03	2.16	0.10	
*c14320f*	*GQ0178_D07*	transcription factor	5	4	height	6	0.02	1.70	0.03	TWM
				4	height	7	0.03	1.67	0.04	
				3	height	12	0.01	2.40	0.10	
*c04312b*	*GQ02754_G07*	C2H2 zinc finger	6	4	height	6	0.02	1.85	0.03	-
				4	height	7	0.02	1.72	0.04	
*c04554n*	*GQ0201_E19*	MYB (10)	7	4	height	6	0.03	1.69	0.03	MAT*, TCM*
				4	height	7	0.05	1.41	0.05	
*c04671m*	*GQ03310_H09*	PLATZ	7	3	budset	7	0.06	0.65	0.08	TCM*
*c07142s*	*GQ0171_A23*	GASA	7	4	budset	8	0.05	1.42	0.08	-
*c08080a*	*GQ0082_F08*	MYB (5)	7	4	budset	8	0.09	1.16	0.10	TWM*, DGD*
				3	budset	7	0.05	0.12	0.08	
*c08085m*	*GQ03515_N01*	Scarecrow-like	7	4	height	7	0.06	0.83	0.07	-
				3	budset	7	0.09	0.17	0.08	
				4	height	6	0.10	1.07	0.09	
*c13572n*	*GQ0178_D07*	Homeobox	7	4	height	6	0.02	1.86	0.03	MAT*, TWM*, TCM*
				4	height	7	0.03	1.67	0.04	
				3	budset	7	0.08	0.34	0.10	
*c15051g*	*GQ0195_G17*	NAM	8	3	budset	7	0.06	0.19	0.10	-
*c03650g*	*GQ03503_G05*	B-box zinc family	9	4	budset	8	0.04	1.50	0.08	TCM*, MAT**
				4	height	6	0.04	1.47	0.05	
				3	budset	7	0.04	0.36	0.08	
*c05937s*	*GQ0207_N03*	Chaperone DnaJ Heat	9	3	height	6	0.01	1.29	0.06	DGD*, TWM*, PWM*
		Shock Prot.		3	height	7	0.02	1.12	0.03	
				3	height	12	0.04	0.99	0.01	
*c09063f*	*GQ02825_P07*	PATATIN	9	4	budset	8	0.08	1.21	0.10	TCM*
				4	height	7	0.10	0.63	0.10	
*c13855n*	*GQ0255_K05*	MYB (11)	9	4	budset	8	0.03	1.65	0.08	MAT***, TCM*, TWM***
*c02623e*	*GQ03208_E14*	HLH	10	4	budset	8	0.06	1.38	0.08	-
*c09573a*	*GQ02904_H04*	HTH	11	4	height	7	0.06	0.84	0.10	-
*c05535s*	*GQ0206_M13*	WRKY	12	4	height	7	0.05	0.90	0.07	-
*c07602a*	*GQ0013_G12*	MYB domain	12	3	height	6	0.01	0.85	0.06	-
*c15328f*	*GQ0071_N15*	MYB	12	4	budset	7	0.03	1.71	0.08	-
				4	budset	8	0.00	2.77	0.03	

^a ^Gene ID follows the nomenclature of the white spruce gene catalogue GCAT [39].

^b ^LG: Linkage group following the nomenclature of [31].

^c ^Site numbers as indicated on Figure 1.

^d ^PVE: Proportion of variance explained.

^e ^Climatic factors significantly correlated with the allele frequency variation of significant gene SNPs: PWM: precipitation of the wettest month; AAP: annual amount of precipitation; TWM: temperature of the warmest month; DGD: annual number of degree-days above 5°C; TCM: temperature of the coldest month; MAT: mean annual temperature. *: *P* ≤ 0.05; **: *P* ≤ 0.01; ***:*P* ≤ 0.005.

**Figure 3:**
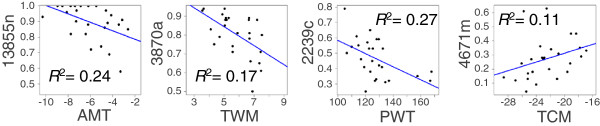
**Examples of linear regressions between the allele frequency of SNPs significantly associated with trait variation and climatic variables of population origins. **MAT, mean annual temperature; TWM, mean temperature of the warmest month; PWT, total amount of precipitation of the wettest month; TCM, mean temperature of the coldest month.

Gene nomenclature conformed to the most recent white spruce gene catalogue (GCAT, [39], Table 2), and the gene SNPs that had been significantly associated with trait variations were spread over the 12 chromosomes of black spruce [31], except for chromosome # 2. Comparison between association test and QTL results showed that 17 SNPs that had been identified using the association tests were also located in QTLs that were delineated in the present work, including seven SNPs related to the same character. The associated SNPs that had been detected were representative of a large array of gene families, including more frequently occurring families such as MYB and AP2, and more interestingly, families such as Chaperone DNAj proteins, which are already known for their role in reactions to abiotic stress (Table 2). A total of 17 SNPs that were associated with adaptive trait variations displayed significant correlations between allele frequency variation and climatic factors for the population origins of the tested material (Table 2, see Figure 3), of which four were highly significant (*P* < 0.005).

## Discussion

### Quantitative trait loci

A total of 22 regions of the *P. mariana* genome were detected as being potentially involved in budset and growth variation, which is lower than the 52 QTLs that had been found in the congeneric white spruce for each of these traits by Pelgas *et al.*[13] which used two pedigrees and larger sets of progeny. A relatively small number of progeny (283) was surveyed in the present study and only one biparental family was used to map QTLs. Given that the progeny were not clonally replicated, in contrast to the study conducted by Pelgas *et al.*[13], it was not expected to identify as many QTLs with as much precision. However, the main goal of the present QTL survey was to obtain an exploratory overview of the genomic architecture of adaptive traits in black spruce for comparative purposes.

Trait variations were not normally distributed, so that the guidelines of Asins *et al.*[40] were followed and, consequently, both parametric and non-parametric approaches were used to identify QTLs. The three different methods yielded overlapping complementary results. The non-parametric methods (nIM and K-W tests) were notably more efficient in QTL detection than the parametric approach (IM), despite their expected lower power to detect QTLs. However, the application of IM still yielded valuable information, such as estimates of the proportions of explained variance. Despite the limitations that were imposed by the available data, four QTLs were repeatedly identified over years or sites, thereby more likely representing genomic regions encompassing polymorphisms that were more central to trait variation. Such a restricted set of QTLs that is shared across years or environments has also been observed in the congeneric *P. glauca*[13].

On average, 8% of the observed phenotypic variance could be explained by a single QTL. This average proportion of explained variance (PVE) seemed low compared to several published values in conifer QTL mapping studies of traits related to growth or adaptive traits [41-43]. Yet, many QTL studies showing high PVE for single QTLs have been performed using interspecific crosses [44-47]. Given that phenotypic variation in such crosses may represent the sum of trait variations originating from both species, QTLs having effects on these adaptive traits would harbour higher PVE estimates accordingly to the average trait differences between the two species. In contrast, the average amplitude of PVE values that were estimated in the present study is consistent with results obtained in other QTL studies on phenology and growth trait variation which had been assessed with intraspecific crosses [13,43,48,49]. Even though such PVE estimates may have been biased upward due to the Beavis effect [50], the congruence with results from other QTL studies relying on larger progeny [13,48], which should be less sensitive to such effect [50], suggests that PVE values were quite accurately estimated in the present study. These results suggested that many QTLs are truly involved in adaptive variation. We noted that three QTL regions were involved in both budset and growth variations, suggesting pleiotropic effects. A similar pattern was also observed among *P. glauca* QTLs for phenology and growth [13]. Such patterns are expected, given that significant positive correlations between growth and budset are generally observed in trees as reported previously in both *P. glauca* and *P. mariana*[25,28]. In other words, the later the budset, the longer the growing season and, consequently, the more growth is generally accumulated.

### SNPs associated with adaptive trait variation

A total of 34 SNPs from as many distinct genes were significantly associated with adaptive trait variations in genetic association tests, representing 65% of the tested candidate SNPs. Such a rate of positives is high, exceeding by more than an order of magnitude the results obtained in other genetic association studies that are based on candidate genes by more than an order of magnitude (*e.g.*[18,20,51]), and where there had been no *a priori* screening of the tested gene polymorphisms. Given the much smaller number of tests that were performed with a limited set of candidate polymorphisms, the issue of multiple testing and potentially high rates of false positives that are associated with intensive testing becomes less of a concern. Although “next-gen” sequencing technologies will allow detection of many more polymorphisms at an affordable cost in the near future, with approaches such as genotyping-by-sequencing [52], statistical issues that are related to high rates of false positives when large numbers of tests are conducted will remain a recurrent theme. As shown here, a candidate SNP approach should contribute to reducing these concerns and the number of tests to be performed, especially when quantitative characters implicating large numbers of genes are considered, or for organisms characterised by very large genomes such as conifers [53]. The outlier analysis and bulk segregant analysis of sequence signatures that were used to identify candidate SNPs were quite efficient in reducing the number of candidate SNPs to be tested by more than an order of magnitude, in both cases (see Methods). The several hundred genes that were involved in the sequencing and preliminary screening procedure for segregating SNPs between opposite pools of extreme individuals represented quite a large number of screened candidate genes, given that we relied on traditional PCR and Sanger sequencing to discover segregating SNPs. New methods relying on massive sequencing of the gene space that use gene sequence capture approaches and next-gen sequencing should make it easier to discover much larger numbers of candidate polymorphisms segregating for a variety of phenotypic characters. In various spruce species, such strategies are currently employed, which involve gene capture and sequencing for a variety of pools using oligo arrays that represent most of the transcriptome of the spruce genome [39].

While screening for candidate gene SNPs was conducted using bulk segregant analysis when plants were 6- and 7-years old, more SNPs were found to be significant in association testing around these ages (6-, 7- or 8-years old) than at a later age of 12 years (Table 2). This trend suggests that different genes and SNPs (and physiological processes) could be involved at different ages, as the transition from juvenile to mature adult is taking place. This scenario is additionally supported by the fact that data from 12-year-old plants were not used in the screening procedure to identify candidate gene SNPs from pool segregant analysis, and that the number of significant genetic association tests was the lowest for field data at that age. A similar trend was not observed in a QTL study of adaptive characters that had been assessed over several years in *P. glauca*, probably because the trees were still at the juvenile stage of development [13]. In the present study, genetic correlations between characters at age 12 and ages 6, 7 and 8 years were generally modest for both sites (data not shown), further pointing to partially different sets of genes and polymorphisms involved. These modest correlations are consistent with previously published estimates from a clonal test in *P. mariana*[54].

In our study, each SNP that was associated with trait variation explained a low proportion of variance (PVE ~ 1.25%), which significantly differed between the two study sites. Average PVE was generally higher for site #4 than for site #3. Site #4 was characterised by harsher climatic conditions at higher altitude (Additional file 1: Table S1), with freezing temperatures occurring earlier in the season than at site #3, the latter site being located at lower elevation and lower latitude. Since budset timing is also significantly related to temperature conditions [23], it was likely that quantitative trait differences between adapted and maladapted trees would have been amplified on the site with harsher climatic conditions, leading to greater ability in detecting significant associations. With respect to this likely trend, the variance in quantitative traits was generally greater on the site with harsher climatic variation (data not shown).

The low PVE values that were observed with significant SNPs suggested that a large number of adaptive polymorphisms from various genes and gene families are involved in the genetic control of adaptation. These low PVE values were not surprising given that similar values have been observed in genetic association studies involving other tree species (3% in [15]; <1% in [55]; 3% in [17]; 0.7-5.4% in [18]; 3-5% in [20]). This trend was consistent with that found for QTLs of the same characters (although lower PVE values were observed in association testing), and in agreement with quantitative genetics theory, which predicts that genetic variation in these quantitative traits is controlled by many genes, each exerting small effects [56,57]. Because linkage disequilibrium usually decays rapidly within gene limits in natural populations of boreal spruces [10,11], the co-occurrence of several physically linked adaptive polymorphisms that would explain a higher proportion of the variance was unlikely.

Several lines of evidence supported the involvement of these significant SNPs in adaptation. First, half of these polymorphisms displayed allele frequency variation significantly correlated with climatic variations (Table 2, Figure 3), hence corroborating, with an extensive population sampling, the trends that had been previously observed in black spruce molecular adaptation studies [26]. In the absence of population structure, these correlations suggested that the alleles are under divergent selection. However, the remaining SNPs should not be considered as false positives given that variation in SNP frequencies may not follow a linear distribution [12]. Although the distribution of an adaptive trait is expected to follow the selective environmental variable, adaptive allele frequency variation along the environmental gradient may follow various and sometimes barely predictable distributions given the complexity of interacting genes and alleles that likely govern adaptive trait variation [12,42].

This idea of complexity of gene and allelic interactions is also reinforced by the large diversity of families and functions observed among the genes that were found to harbour significant SNPs. We noted that 53% of significant SNPs belonged to gene families previously found to harbour genetic polymorphisms and expression patterns that were related to stress resistance and adaptation in other conifers, trees or plant model organisms. For instance, genes of the MYB family have already been reported in several studies as transcription factors that are involved in growth control, response to cold stress and organ differentiation, and related to budset in *P. glauca*[20,58-60]. In addition, MYB members have also been discovered in genetic association studies of cold hardiness in *Pseudotsuga menziensii* var. *menziesii*[17] and *Picea sitchensis*[18]. A SNP in a BAM gene was also associated with ring growth variation in a previous association study in *P. glauca*[20]. As budset timing and cold resistance are preceded by growth cessation [23], we expected to find genes regulating growth development. As was the case for genes from the AP2 family, which have been identified previously in the gene network activating plant freezing tolerance in *Arabidopsis*[61] and *Triticum aestivum*[62]. Similarly, variation in the expression of genes from the C3HC4 RING family has been observed under drought and low temperature stress in *Arabidopsis* and rice [63,64]. In the latter species (*Oryza sativa* L.), a protein of the WRKY family was also found to be activated by a Heat Shock protein under drought stress [65]. Dnaj Heat Shock proteins are produced under diverse abiotic stresses [66] and both WRKY and Heat Shock families were represented by a gene SNP that was associated with height variation in the present study. Among other significant gene SNPs that were found in the present study, the NAC, LEA, PATATIN and RAP2 gene families were also represented. NAC proteins are over-expressed in response to drought and salinity stresses in rice [67], as well as in drought responses of *Arabidopsis*[68]. In addition, several LEA genes are known to improve resistance to dehydration and freezing temperatures in plants [69] and were reported in a drought adaptation study in *Pinus pinaster* Aiton [70]. It should be also noted that a PATATIN gene has been found to be stimulated by drought stress in *Vigna unguiculata* (L.) Walp. and a RAP2 gene was found to be up-regulated by drought stress in *Arabidopsis*. Hence, a wide variety of gene families was found, indicative of the large number of physiological processes involved in budset and growth.

Of the 34 SNPs significantly associated with adaptive trait variation, seven were also located in a QTL for the same character. Given the known relationships between budset timing, the length of the growing season and growth (see above), it is plausible that several SNPs that were associated to one trait were located in a QTL for the other trait. Considering such cases, 17 significant SNPs (50%) were located in QTLs. Such corroboration would support the identified polymorphisms as true positives. Furthermore, the two analytical approaches should not be expected to be congruent but complementary. QTL mapping permits the identification of polymorphisms involved in trait variation in one or few pedigree populations, thereby reducing background genetic variations as well as the number and complexity of interacting alleles, while genetic association testing permits the identification of polymorphisms of more general value over a wider range of genetic backgrounds.

In total, 65% of the significant SNPs that had been identified by genetic association tests were corroborated by additional indirect evidence of various sources, as indicated above (relationships with climatic factors, adaptation QTLs and key gene families). This evidence highlights their relevance for possible *in situ* monitoring of genetic variation for adaptation or for inclusion in marker-aided breeding selection schemes. The remaining gene SNPs could still represent polymorphisms of high adaptive value, even though additional research would be required to identify their specific role in adaptation.

## Conclusion

In this report, 22 unique QTL regions and 34 gene SNPs that were involved in budset timing and tree height variation were identified using QTL mapping and genetic association testing. The significant polymorphisms may not be causative of the quantitative variation observed, but they may be linked to neighbouring true adaptive SNPs [8]. Although this possibility cannot be ruled out, linkage disequilibrium is so low in natural populations of conifers [10] that the true adaptive polymorphism would likely lie within the same gene. Consequently, even if an identified SNP may not be the “true” adaptive SNP, it might remain a valuable marker for various genetics applications, such as for monitoring natural adaptation or for marker-assisted selection related to assisted migration. In addition, QTL mapping and genetic association tests were conducted using samples from experimental sites with contrasting environmental conditions, which revealed a few genomic regions and SNPs detected in both sets of conditions. These loci and their SNPs are of great interest for the above-mentioned applications since they appear to have an effect in a broad set of environmental conditions.

The low estimates of PVE that were obtained and the large diversity of families and functions of genes carrying significant SNPs indicated that a large number of polymorphisms and genes are involved in adaptive trait variation, implying that these SNPs should be simultaneously considered in predicting a significant proportion of observed variation in adaptive traits [71]. Given the large size of conifer genomes and their low LD in natural populations, an extensive number of markers would be needed to systematically cover the genome and sample most of the quantitative trait nucleotides (QTNs), including those within intergenic regions that possibly play a role in quantitative trait variation [72]. To circumvent this issue, it has been proposed to reduce effective population size of the targeted populations in order to increase linkage disequilibrium between markers and QTLs and capture most of the QTLs, a strategy generally known as genomic selection [73-75]. With such strategies, the number of markers required to predict accurately the phenotype could be reduced to a few per centimorgan for effective population sizes of tens of individuals [76]. Even though this more anonymous approach is highly useful for breeders and perhaps for assisted migration, it would scarcely shed light regarding the nature of adaptive genes and polymorphisms and be ill-adapted for monitoring purposes in natural populations. Therefore, we anticipate that additional efforts will be required to understand more completely the genetic bases of complex adaptations. To this end, resequencing of the transcriptome of black spruce has been initiated and the simultaneous genotyping and detection of gene SNPs that segregate for adaptive characters in natural populations will be pursued.

## Methods

### QTL mapping analyses

The QTL analysis was conducted using a backcross family (#9920002: ♀11307-03 [♀83 x ♂425] × ♂425) of 283 individuals already used to map the genome of black spruce [30,31]. The genome of black spruce contains 12 chromosomes corresponding to as many different linkage groups, as is the case for most other Pinaceae and all spruces that have been mapped so far [10,30,31,77]. The controlled cross used for mapping was performed in 1999. The progeny was seeded in greenhouse in 2000 and planted in 2002 on site #1 (46°38′N, 72°00′W; 35 m elevation; Figure 1). Clones (rooted cuttings in 2002) of these individuals were subsequently planted in 2004 on site #2 (47°45′N, 70°50′W; 792 m elevation; Figure 1), which was characterised by harsher climatic conditions at higher altitude (Additional file 1: Table S1). However, no clonal replicates were available on each site. Budset timing was assessed weekly between mid-July and October (~13 measurements) and scored following a three-stage morphological index as in [25]: (0) absence of terminal bud, (1) presence of a white terminal bud, (2) brownish terminal bud, and (3) terminal bud with scales ready to endure winter conditions. For each tree, these scores were averaged over the measurement period. Growth was assessed by measuring terminal shoot elongation of each tree at the end of the growing season. These measurements were collected on site #1 for 254 and 244 progeny in 2006 and 2007, respectively and on site #2 for 155 and 142 progeny for the same years (differences in sample size between years was due to mortality).

As suggested for traits not normally distributed [40], the QTL analysis was conducted following parametric and non-parametric approaches. First, a traditional interval mapping approach was applied to the transformed data to fit the normal distribution required for this method. It was implemented in MapQTL 5.0 [78], and QTLs having LOD scores greater than a threshold value that had been determined by a permutation test were retained (1000 permutations were applied at the genome-wide level or each linkage group separately). Second, non-parametric interval mapping was applied to untransformed phenotypic data using the R/qtl package [79] for R (R Development Core Team, 2011). As was the case for the parametric interval mapping approach, the minimum LOD score threshold was determined using a permutation test that allowed the identification of significant QTLs. Finally, a Kruskal-Wallis test was applied to phenotypic data to test each marker, keeping in mind that this represented a multiple testing procedure. Loci repeatedly identified over years, sites and methods were considered as QTLs of putative interest.

### Genetic association testing

#### Candidate gene SNPs

To reduce the number of association tests, a list of candidate gene SNPs was determined from the results of a previous study of gene SNPs in relation to temperature and precipitation variation in black spruce [26], together with the results of comparisons among gene sequences of individuals presenting extreme phenotypes for budset timing or tree height using bulk segregants analysis. By assessing budset timing as described above and measuring tree height at ages six and seven in a provenance/progeny test of black spruce that had been initiated in 1999 [25], DNA pools of the 10 individuals that presented extreme values for these two variables were assembled and sequenced as in Pelgas *et al.*[80], at a rate of no more than one tree per open-pollinated family present in the provenance/progeny test. For each year, one pool of the 10 individuals displaying the earliest budset timings (i.e., the highest scores) and one pool of the 10 individuals showing the latest budset timings (i.e., the lowest scores) were assembled, together with one pool with the 10 shortest individuals and one pool with the 10 tallest individuals. Sanger sequencing was conducted on the assembled pools using a total of 434 primer pairs, each representing a different gene and covering a large array of families and functions (Additional 2: file Table: S2). Afterwards, comparisons of the sequences that were based on the relative intensity of nucleotide signatures in trace files were made according to the criteria of Pelgas *et al.*[80]. A total of 41 candidate gene SNPs segregating between contrasting pools were identified and classified as candidate polymorphisms involved in budset timing or height growth variation or both (see Results). An additional set of 26 candidate gene SNPs were also identified from previous outlier detection studies related to variation in temperature or precipitation in the same geographic area for black spruce and based on a largely similar set of genes [26]. These 26 outlier SNPs were identified from a total of 583 SNPs representing 313 genes that had been successfully genotyped in natural populations [26]. Many of these SNPs have also been shown to be related to temperature and precipitation variation at the range-wide level [12]. Because 15 SNPs were identified by both methods (outlier detection and bulk segregant analysis), the final set contained 52 distinct candidate SNPs representing 51 genes for formal genetic association testing (see Results).

#### Sampling and phenotypic measurements

The genetic association study was based on a provenance/progeny test established on two different sites in 1999 and which represented natural populations from the province of Quebec, eastern Canada (Figure 1). The provenance/progeny test was composed of 30 populations, each represented by three open-pollinated families [25]. Given the potential natural occurrence of introgression between red spruce (*Picea rubens* Sarg.) and black spruce in the region close to the sympatric zone between these two species [81], four populations presenting trees with genetic markers specific to red spruce [82,83] had to be discarded.

A total of 991 individuals were sampled on site #3 (45°48′N, 70°44′W, Figure 1), which was characterised by relatively warmer conditions at low altitude (200 m; Additional file 1: Table S1), and 492 individuals on site #4 (47°15′N, 71°10′W, Figure 1), which was subject to relatively harsher climatic conditions at higher altitude (770 m; Additional file 1: Table S1). For each individual, budset timing and tree height were assessed at 6 years of age in 2005 on site #3, at 7 years of age in 2006 on both sites, and at 8 years of age in 2007 on site #4. Additionally, tree height was measured on both sites at the age of 12 (2011). Budset timing was assessed as described for QTL mapping (see above). For each tree, needles were collected and DNA was extracted using the Nucleospin extraction kit, following manufacturer’s instructions (Clontech Laboratories Inc, Foster City, CA, USA).

#### Control SNPs, candidate SNPs and genotyping for genetic association tests

To ensure efficient performance of the genetic association tests, control SNPs were required to estimate the degree of covariation among individuals (kinship, see below). A subset of 36 control SNPs from as many distinct genes were thus chosen among SNPs that had not been identified as related to climatic factors in a previous outlier identification study in the same region [26].

For a total of 88 SNPs that encompassed 52 candidate SNPs (see section Results), 1483 individuals were genotyped using Sequenom iPLEX Gold genotyping technology, which is particularly suited to genotyping large numbers of samples for a limited number of SNPs. Based on multiplexed PCR, this technique uses mass spectrometry for product separation and detection (http://www.sequenom.com), thereby allowing genotypes for 36 SNPs to be obtained at a time. Sequenom genotyping was conducted under the supervision of A. Montpetit at the Genome Québec Innovation Centre (McGill University, Montreal). Given the complexity of the multiplexed PCR assays, five candidate SNPs could not be genotyped using this technology. For each of these, genotyping was done using endpoint genotyping analysis, which uses two allele-specific probes (TaqMan) that are labelled with different fluorescence dyes and employed in a qPCR analyser (LightCycler 480, Roche Diagnostic). Genotypes were then obtained by measuring the intensity distribution of dyes detected by the same instrument. A call rate > 90% and a minor allele-frequency > 2% were applied as criteria for quality control.

#### Genetic association tests

The most common bias in genetic association testing is the possibility that individuals harbour common alleles owing to identity by descent (IBD) and not related to similar phenotypic values. Statistical methods have been subsequently developed to take into account the degree of genetic relatedness among individuals, which allow adjusted association tests to be performed such as the approach described by Yu *et al.*[37]. With this method, pairwise relative kinship coefficients (*K*) are estimated using control markers and then incorporated into a unified mixed-model to correct for variation attributed to IBD. In the present study, pairwise kinship coefficients were estimated using the method of Loiselle *et al.*[32], as implemented in the program SPAGeDI [84], and using the 31 successfully genotyped control SNPs (see results). Then, the effect of each SNP was tested using analysis of variance of each adaptive trait among genotypic classes as implemented in TASSEL [37] and following the mixed-model:

(1) Y=Sα+Zu+e

where α is a vector of SNP effects, u is a vector of polygene background effects, e is a vector of residual effects, and *S* and *Z* are incidence matrices of 0 s and 1 s relating Y to α and u, respectively. The variances of the random effects were assumed to be:

(2) $$\text{Var}\left(u\right)\;=\;2\text{KVg}\;\text{and}\;\text{Var}\left(e\right)\;=\;RV_{R}$$

where *K* is a n × n matrix of relative kinship coefficients that were estimated using SPAGeDI; R is a n × n matrix in which the off-diagonal elements are 0 s and the diagonal ones are the reciprocals of the number of observations for which each genotypic data point was obtained; *Vg* is the genetic variance; and *V*_*R*_ is the residual variance [37]. These genetic association analyses were conducted separately for each study site, given their environmental and climatic differences and possible genotype × environment interactions.

When population genetic structure is significant and high, it is usually taken into account when performing association genetic tests [17,18]. The population genetic structure of *P. mariana* in the sampled region has been previously investigated using a number of approaches and genetic markers (RAPDs, ESTPs, gene SNPs, cpSSRs), repeatedly resulting in average *F*_ST_ values that were close to zero, large gene flow estimates, and no discernible genetic structure [12,26,34-36,85]. The populations that were sampled also belong to the same historical lineage [12,43]. Despite this multiple evidence, to further ensure that no hidden genetic structure existed among the sampled populations, we investigated population structure using the 31 control SNPs which were assessed for all individuals, and by using STRUCTURE [33]. Burn-in and running lengths were set to 100,000 iterations, and the number of clusters of individuals displaying the highest probability was considered optimal.

SNPs that were found to be significantly associated with adaptive trait variation were checked for gene physical locations, gene families and possible amino-acid changes induced by nucleotide substitutions. In addition, climatic conditions were obtained for each population of origin (Additional file 3: Table S3), using an extrapolation model, based on 1971–2000 measurements from nearby weather stations, and implemented in BioSIM [86,87]. Then, *a posteriori* correlations among adaptive allele frequencies and climatic variables that were related to temperature and precipitation of population origins were checked for potential relationships. The climatic variables that were investigated included mean annual temperature, total amount of precipitation, mean temperature of the coldest month, mean temperature of the warmest month, the amount of precipitation of the wettest month, the amount of precipitation of the driest month, and the annual number of degree-days ≥ 5°C.

## Competing interests

The authors declare that they have no competing interests.

## Authors’ contributions

JP, MD, JBe: provenance tests, phenotypic measurements, sampling and climatic data gathering; FG: sampling, DNA extractions, genotyping supervision and data quality. JP, BP and NI: QTL mapping strategy and analyses; JP, JBo and JBe: genetic association test design and analyses. MD, NI, JBe and JBo: study funding. JP and JBo: manuscript preparation. All authors read and approved the final manuscript.

## Supplementary Material

Additional 1: Table S1 Climatic conditions for each experimental site depicted in Figure 1. Click here for file

Additional 2: Table S2 List of genes and primer pairs used for DNA pool sequencing of bulk segregant samples. Click here for file

Additional 3: Table S3 List of geographical coordinates and weather conditions for the locations of sampled populations. Click here for file
