# Supplementary material for: The genomic architecture and association genetics of adaptive characters using a candidate SNP approach in boreal black spruce
Source: BMC Genomics. 2013 Jun 1;14:368. doi: 10.1186/1471-2164-14-368 (PMC3674900; doi:10.1186/1471-2164-14-368)
Supplement: Additional 1: Table S1 — Climatic conditions for each experimental site depicted in Figure 1. [file 1471-2164-14-368-S1.docx]

Supplementary table S1 Climatic conditions for each experimental site depicted in Fig. 1

| Experimental  Site | Latitude  (^o^N) | Longitude  (^o^W) | Mean annual temperature  (^o^C) | Total annual precipitation (mm of water) | Degree-days > 5°C |
| --- | --- | --- | --- | --- | --- |
| 1 | 46.38 | 72.00 | 4.40 | 994.59 | 1763.20 |
| 2 | 47.45 | 70.50 | -0.32 | 1375.18 | 986.98 |
| 3 | 45.48 | 70.44 | 3.51 | 994.04 | 1470.40 |
| 4 | 47.15 | 71.10 | -0.38 | 1616.49 | 940.96 |
