# Supplementary material for: The genomic architecture and association genetics of adaptive characters using a candidate SNP approach in boreal black spruce
Source: BMC Genomics. 2013 Jun 1;14:368. doi: 10.1186/1471-2164-14-368 (PMC3674900; doi:10.1186/1471-2164-14-368)
Supplement: Additional 3: Table S3 — List of geographical coordinates and weather conditions for the locations of sampled populations. [file 1471-2164-14-368-S3.docx]

Supplementary table S3 List of geographical coordinates and weather conditions for the locations of sampled populations.

| Population | Longitude (°W) | Latitude (°N) | Elevation (m) | Mean annual temperature (°C) | Mean temperature of the coldest month (°C) | Mean temperature of the warmest month (°C) | Annual precipitation (mm of water) | Precipitation of the driest month (mm of water) | Precipitation of the wettest month (mm of water) | Number of annual degree-days > 5°c |
| --- | --- | --- | --- | --- | --- | --- | --- | --- | --- | --- |
| 47 | -74.22 | 49.7 | 396 | -1.53 | -19.42 | 16.36 | 86.94 | 46.04 | 127.83 | 86.94 |
| 325 | -73.63 | 50.45 | 365 | -2.56 | -20.76 | 15.64 | 83.81 | 39.58 | 128.05 | 83.81 |
| 326 | -73.45 | 49.03 | 380 | -1.06 | -18.53 | 16.41 | 89.82 | 49.94 | 129.71 | 89.82 |
| 329 | -71.2 | 47.87 | 810 | -1.20 | -17.24 | 14.84 | 125.32 | 82.97 | 167.66 | 125.32 |
| 330 | -65.42 | 48.92 | 610 | 1.32 | -13.69 | 16.34 | 100.33 | 78.07 | 122.60 | 100.33 |
| 331 | -64.88 | 48.4 | 120 | 2.18 | -12.08 | 16.44 | 92.65 | 68.63 | 116.68 | 92.65 |
| 332 | -67.12 | 48.5 | 245 | 1.28 | -14.45 | 17.00 | 92.91 | 60.45 | 125.37 | 92.91 |
| 333 | -77.75 | 49.62 | 270 | -1.72 | -19.59 | 16.14 | 77.64 | 33.88 | 121.39 | 77.64 |
| 336 | -76.95 | 48.37 | 365 | -1.21 | -18.85 | 16.43 | 87.04 | 42.12 | 131.96 | 87.04 |
| 338 | -76.55 | 47.08 | 360 | 0.74 | -16.45 | 17.92 | 80.06 | 50.12 | 110.00 | 80.06 |
| 341 | -67.15 | 50.13 | 145 | -0.36 | -16.50 | 15.78 | 94.67 | 65.92 | 123.43 | 94.67 |
| 342 | -68.77 | 50.67 | 430 | -1.75 | -19.08 | 15.59 | 92.57 | 52.64 | 132.51 | 92.57 |
| 343 | -68.83 | 49.2 | 74 | 1.07 | -15.41 | 17.55 | 79.50 | 54.08 | 104.91 | 79.50 |
| 345 | -69.13 | 48.93 | 120 | 1.20 | -15.25 | 17.66 | 90.32 | 61.56 | 119.09 | 90.32 |
| 347 | -73.1 | 47.3 | 360 | 0.59 | -15.98 | 17.15 | 98.00 | 59.11 | 136.88 | 98.00 |
| 352 | -71.3 | 49.6 | 185 | -1.13 | -19.18 | 16.92 | 89.23 | 44.08 | 134.39 | 89.23 |
| 353 | -70.93 | 48.2 | 245 | 0.34 | -16.72 | 17.39 | 88.40 | 53.45 | 123.34 | 88.40 |
| 354 | -70.1 | 48.83 | 730 | -2.86 | -21.42 | 15.69 | 112.26 | 66.88 | 157.64 | 112.26 |
| 355 | -63.37 | 49.63 | 185 | 0.56 | -13.89 | 15.01 | 85.42 | 55.90 | 114.95 | 85.42 |
| 356 | -76.68 | 48.6 | 440 | -0.80 | -18.03 | 16.43 | 86.65 | 43.67 | 129.62 | 86.65 |
| 359 | -66.43 | 48.57 | 275 | 1.37 | -13.89 | 16.63 | 94.64 | 64.48 | 124.81 | 94.64 |
| 360 | -64.92 | 48.25 | 170 | 2.55 | -11.82 | 16.92 | 98.63 | 77.23 | 120.03 | 98.63 |
| 361 | -65.85 | 48.48 | 185 | 2.23 | -12.62 | 17.09 | 85.57 | 57.63 | 113.51 | 85.57 |
| 362 | -77.97 | 49.05 | 305 | -1.16 | -18.49 | 16.18 | 79.48 | 32.05 | 126.90 | 79.48 |
| 367 | -76.5 | 46.92 | 305 | 1.24 | -15.72 | 18.20 | 78.63 | 50.78 | 106.48 | 78.63 |
| 443 | -71.23 | 48.03 | 655 | -0.43 | -16.41 | 15.56 | 121.76 | 76.71 | 166.81 | 121.76 |
